# Efficacy of Histone Deacetylase and Estrogen Receptor Inhibition in Breast Cancer Cells Due to Concerted down Regulation of Akt

**DOI:** 10.1371/journal.pone.0068973

**Published:** 2013-07-09

**Authors:** Scott Thomas, K. Ted Thurn, Paromita Raha, Stephanie Chen, Pamela N. Munster

**Affiliations:** Division of Hematology and Oncology, Department of Medicine, University of California San Francisco, San Francisco, California, United States of America; The University of Arizona, United States of America

## Abstract

Hormonal therapy resistance remains a considerable barrier in the treatment of breast cancer. Activation of the Akt-PI3K-mTOR pathway plays an important role in hormonal therapy resistance. Our recent preclinical and clinical studies showed that the addition of a histone deacetylase inhibitor re-sensitized hormonal therapy resistant breast cancer to tamoxifen. As histone deacetylases are key regulators of Akt, we evaluated the effect of combined treatment with the histone deacetylase inhibitor PCI-24781 and tamoxifen on Akt in breast cancer cells. We demonstrate that while both histone deacetylase and estrogen receptor inhibition down regulate AKT mRNA and protein, their concerted effort results in down regulation of AKT activity with induction of cell death. Histone deacetylase inhibition exerts its effect on AKT mRNA through an estrogen receptor-dependent mechanism, primarily down regulating the most abundant isoform AKT1. Although siRNA depletion of AKT modestly induces cell death, when combined with an anti-estrogen, cytotoxicity is significantly enhanced. Thus, histone deacetylase regulation of AKT mRNA is a key mediator of this therapeutic combination and may represent a novel biomarker for predicting response to this regimen.

## Introduction

Breast cancer remains one of the most serious diseases to afflict women, being the most commonly diagnosed malignancy, and second only to lung cancer as the cause of cancer-associated death [1]. For patients with tumors that over-express estrogen receptors (ERs), hormonal therapy reduces the risk of recurrence and improves survival in patients with metastatic disease [2]. Although selective ER modulators, down regulators, and aromatase inhibitors have been used effectively in the postmenopausal setting, tamoxifen remains the only choice for treating premenopausal women who do not wish to suppress their ovarian function chemically or surgically [3]. Nevertheless, the effectiveness of these agents is limited by the development of resistance, arising in nearly 50% of all patients treated with hormonal therapy. Many cellular changes have been suggested as underlying mechanisms for acquired anti-estrogen resistance. These include altered ER expression and ligand independence, down regulating tumor suppressors such as PTEN, and up regulating drivers and their activity, such as Akt [4]. Recent approval of the mTOR inhibitor everolimus suggests that targeting the AKT/mTOR pathway is a successful approach in the setting of hormonal therapy resistance [5].

Studies conducted by our and other groups have demonstrated that when combined with an HDAC inhibitor, the cytotoxic activity of tamoxifen is enhanced in breast cancer cells [6–8]. The increased cytotoxicity is the result of re-directing cells from growth arrest into apoptosis. This is manifested by up regulation of apoptotic drivers such as Bax, and down regulation of apoptotic inhibitors such as Bcl-2, which leads to release of mitochondrial cytochrome C, caspase activation, and cell death [7,8]. Recently, we completed a phase II clinical trial evaluating the combination of the HDAC inhibitor vorinostat with tamoxifen in 43 patients with advanced breast cancer who had previous progressed on aromatase inhibitors [9]. These patients had been heavily pretreated. More than half of the patients had received two or more aromatase inhibitors and adjuvant tamoxifen, and nearly two-thirds had received prior chemotherapy. In 40% of these patients, hormone therapy resistance was reversed and disease was stabilized for > 6 months (21%) or the tumor burden reduced > 30% (19% partial responses). The significance of these findings was illustrated in the control group of a separate trial, where a similar patient population received tamoxifen and no objective responses were observed [10]. Although promising, the limited understanding of the mechanistic underpinnings of this combination prevents the successful pre-selection of patients who are more likely to benefit.

The Akt serine–threonine family of kinases is frequently found over-expressed or hyper-activated in a variety of tumor types, including breast cancers [11–14]. This family of kinases consists of three homologous isoforms (Akt1, Akt2, and Akt3) that function as major effectors of PI3 kinase signaling, regulating a myriad of cellular processes including the promotion of survival, glucose metabolism, proliferation, and protein translation [15]. Akt kinases are recruited to the plasma membrane by their pleckstrin homology domain, where they are phosphorylated and activated by PDK1 and the mTORC2 complex [16,17]. Activated Akt propagates the signal by phosphorylating downstream targets such as the apoptosis promoting BH3-domain protein Bad, the forkhead transcription factor FoxO1, and the kinase GSK-3 beta [18–20].

Previous studies have shown that HDAC inhibition down regulates Akt activity in MCF7 breast cancer cells. This was partly the result of excluding HDACs from PP1 complexes, leading to Akt de-phosphorylation and reduced activity [21]. In turn, the activity of the negatively regulated Akt target, GSK-3 beta remained high, thus driving cyclin D1 ubiquitylation and proteasomal degradation [22,23]. In primary mouse chondrocytes, HDAC3 is linked to Akt activation through the regulation of PH domain and leucine-rich repeat phosphatase 1 expression [24]. These findings raised the possibility that the efficacy of combining HDAC inhibition with an anti-estrogen may be the result of down regulating Akt activity. In the current study, we sought to test this hypothesis. Our findings demonstrate that HDAC and ER inhibition act concertedly to down regulate AKT mRNA, protein and activity in ER-positive breast cancer cells. HDAC inhibitors exert their effect on Akt expression through an ER-dependent mechanism. The extent of AKT down regulation correlates with the degree of cytotoxic synergy observed with combined HDAC and ER inhibition, and Akt depletion is sufficient to increase tamoxifen cytotoxicity. Thus, modulating the relationship between HDACs and AKT may be key to reversing hormone therapy resistance. Furthermore, AKT down regulation may represent a novel biomarker enabling the selection of patients most likely to respond to this novel approach.

## Materials and Methods

### Chemicals and Antibodies

Valproate, trichostatin A, fulvestrant, actinomycin D, and cycloheximide were purchased from Sigma-Aldrich (St. Louis, MO). PCI-24781 was provided by Pharmacyclics Inc. (Sunnyvale, CA). 4-OH-tamoxifen was purchased from Calbiochem (San Diego, CA). Cyclin D1, ER and PR antibodies were purchased from Santa Cruz Biotechnology Inc. (Santa Cruz, CA). Pan-Akt, Akt1, Akt2, Akt3, Akt-P^S473^, Akt-P^S308^, Bax, Bim, FoxO1-P^S2448^, mTOR-P^S256^, and PARP antibodies were purchased from Cell Signaling Technology (Danvers, MA). Cytochrome C antibody was provided with the ApoAlert Cell Fractionation kit (Clontech, Mountain View, CA). GAPDH antibody was purchased from Chemicon (Temecula, CA).

### Cell Culture

All cell lines were purchased from the American Type Culture Collection (Manassas, VA) and verified by short tandem repeat sequencing. Cell lines were maintained in Dulbecco’s Modified Eagle’s Medium (Fisher Scientific, Atlanta, GA) with 10% fetal bovine serum (FBS, Hyclone, Logan, UT), 2 mM glutamine, and 50 unit/mL penicillin and 50 µg/mL streptomycin (Fisher Scientific). Cells were incubated in a humidified atmosphere with 5% CO_2_ at 37°C.

### siRNA Depletion

siRNA duplexes for ER (ID#42835) were purchased from Applied Biosystems (Carlsbad, CA) and SMARTpool siRNAs for AKT1 (L-003000-00-0005) and AKT2 (L-003001-00-0005) were purchased from Thermo Scientific (Dharmacon products, Lafayette, CO). Cells were transfected with siRNA duplexes by nucleofection using the Nucleofector transfection kit according to the manufacturer’s recommendations (Amaxa, Gaithersburg, MD) as previously described [7]. Experiments were conducted as indicated the following day. The *Silencer* negative control 2 siRNA (Applied Biosystems, ID#4613) was used as a nucleofection control, and herein referred to as scramble.

### Cell Proliferation, Viability, and Apoptosis Assays

At the indicated time, cell proliferation was assayed using Celltiter 96 AQueous One solution as per manufacturer’s instructions (Promega, Madison, WI). Cell viability was determined by dye exclusion assay. Briefly, adherent and floating cells were combined and trypan blue solution (Hyclone) was added 1:1 (v/v) and incubated for 2 minutes at room temperature (RT). Viability was measured using a TC10 Automated cell counter (BioRad, Hercules, CA). To assay for apoptosis, cells were harvested and washed in PBS. Cells were cytospun onto slides, stained with the DeadEnd fluorometric TUNEL kit (Promega) and counterstained with DAPI to visualize cell nuclei. Using fluorescent microscopy, cells were scored for TUNEL staining, counting a minimum of 100 cells per experiment. All treatments were conducted in triplicate.

### Cell Fractionation

The cytoplasmic fraction was separated from mitochondria using the ApoAlert Cell Fractionation Kit according to the manufacturer’s protocol (Clontech). Briefly, ~3x10^7^ cells were harvested, washed with ice-cold PBS, followed by washing with Cell Wash Buffer. Washed cells were incubated in Cell Fractionation buffer on ice for 10 minutes, dounce homogenized, and centrifuged at 700 x *g* for 10 minutes at 4°C to pellet debris. The supernatant was centrifuged at 10000 x *g* for 25 minutes at 4°C to pellet mitochondria. The supernatant was collected and saved as the cytoplasmic fraction. Cytoplasmic cytochrome C was quantified by western blot.

### Western Blot Analysis

Cells were harvested, washed with ice-cold PBS, and solubilized using SDS lysis buffer (0.1% SDS, 1% triton X-100, 50 mM Tris-HCl pH 7.4, 150 mM NaCl, 10% glycerol, Halt Protease and Phosphatase inhibitor cocktail (Thermo Scientific), and 1 mM PMSF) by passing the cell suspension through a 20-gauge needle. Debris was cleared from samples by centrifuging for 15 minutes at 14000 x *g*. Lysate protein concentration was determined by Bradford assay (BioRad). Western blot analysis was conducted as previously described [7].

### Akt Kinase Assay

Akt kinase activity was assessed using the non-radioactive Akt kinase assay kit according to the manufacturer’s instructions (Cell Signaling Technology). Briefly, following treatment, cells were harvested, suspended in provided lysis buffer, sonicated, and centrifuged (14000 x *g*, 15 minutes, at 4°C) to clear debris. Phosphorylated Akt was immunoprecipitated by incubating lysate with immobilized phosho-Akt antibody beads overnight at 4°C. Beads were then washed twice in lysis buffer, once with kinase buffer, and incubated with a GSK-3 fusion protein substrate and ATP in kinase buffer at 30°C for 30 minutes. GSK-3 fusion protein phosphorylation was evaluated by western blot.

### Messenger-RNA Expression Analysis

Cells were seeded to 6-well plates at 3x10^5^ cells/well and treated as indicated the following day. Addition of 5 µg/mL actinomycin D and 10 µg/mL cycloheximide were used to inhibit transcription and translation, respectively. At the indicated times, cells were harvested and washed with ice-cold PBS. For mRNA purification, the Qiagen RNeasy kit (Valencia, CA) was used according to manufacturer’s instructions. Taqman expression assays for AKT1 (ID# Hs00178289_m1), AKT2 (ID# Hs01086102_m1), AKT3 (ID# Hs00987350_m1), ESR1 (ID# Hs00174860_m1), and beta-glucuronidase (ID# Hs00939627_m1) were purchased from Applied Biosystems (Carlsbad, CA). Following quantification using a Nanodrop spectrophotometer (Thermo Scientific), mRNA was reverse transcribed. Resultant cDNA (5 nM) was quantified using the appropriate Taqman expression assay (500 nM forward and reverse primers and 200 nM of probe) with an ABI 7900HT PCR system (Applied Biosystems). Each experimental treatment was conducted in triplicate and each singlet was assayed by Taqman expression in triplicate. Expression was measured as “cycles to threshold” (CTs). Expression of mRNA was normalized to beta-glucuronidase expression.

### Flow cytometry

Cell cycle was determined using the Accuri 6 flow cytometer (BD Biosciences). MCF7 cells were treated as indicated, harvested, washed, and fixed in 3% paraformaldehyde. Fixed cells were treated with 2 mg RNAse A, 0.1% triton-X100, 20 µg/mL propidium iodide and incubated at 37°C for 15 minutes. For each sample, 10,000 events were captured. The percentage of G1, S, and G2/M cells was determined using ModFit LT software (Verity Software House).

### Statistical Analysis

The significance of differences between data sets were determined using the Student’s *t* test with Microsoft excel software.

## Results

### The novel HDAC inhibitor PCI-24781 and tamoxifen concertedly down regulate Akt protein and activity

**Figure 1:**
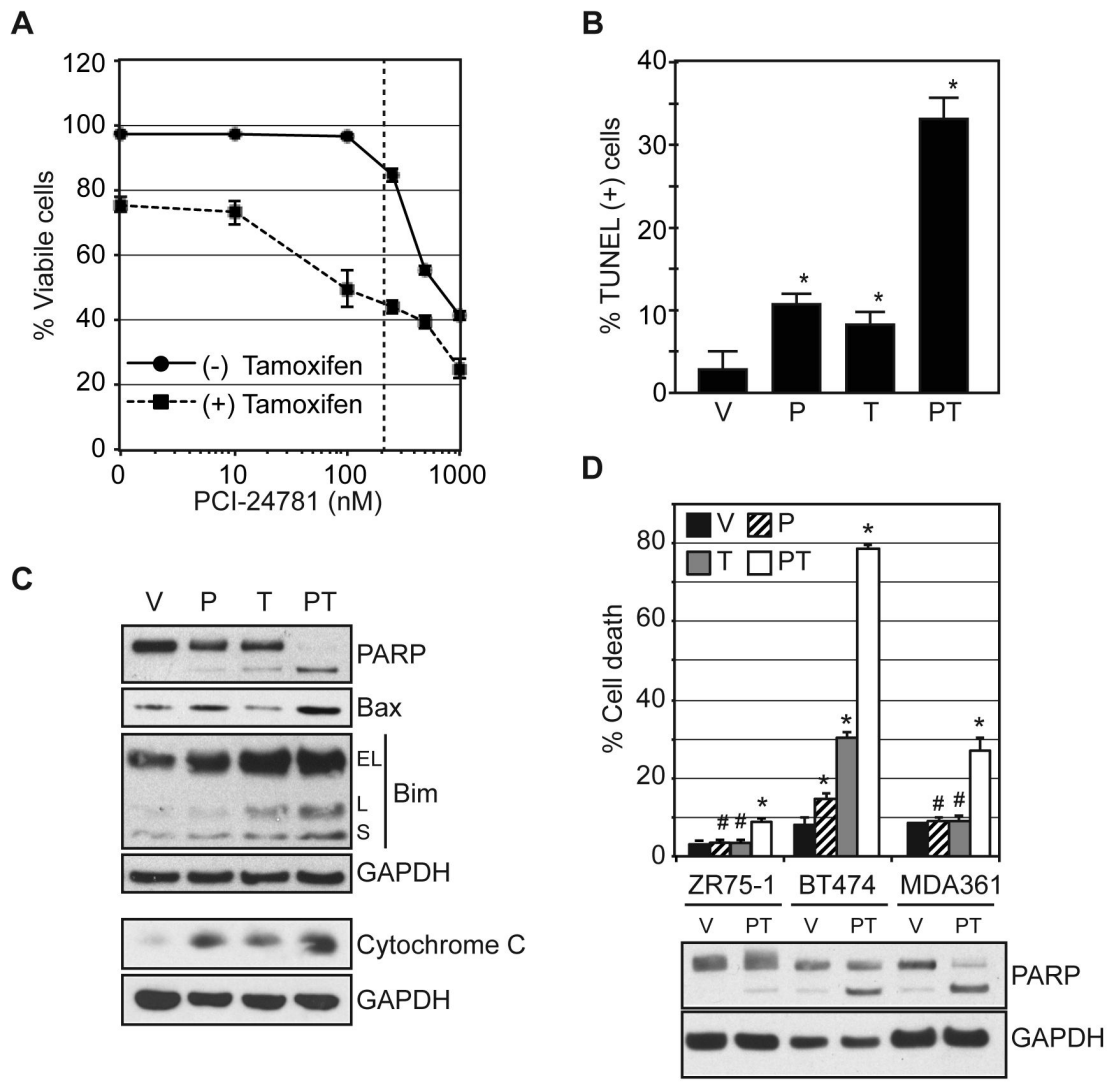
The HDAC inhibitor PCI-24781 potentiates tamoxifen by inducing mitochondrial-mediated apoptosis. (**A**) MCF-7 cells were treated with increasing concentrations of PCI-24781 and with or without 10 µM OH-tamoxifen for 72 hours and assayed for viability. The dotted line indicates maximal achievable serum levels in patients [26]. MCF-7 cells were treated with vehicle (V), 0.1 µM PCI-24781 (P), 10 µM OH-tamoxifen (T), or the combination (PT) for 72 h. Cells were evaluated by western blotting for expression of apoptotic BH3 family member proteins, cytoplasmic cytochrome C, and PARP cleavage (**C**) and by microscopy for TUNEL staining (**B**). (**D**) The indicated cell lines were treated with vehicle (V), 0.1 µM PCI-24781 (P), 10 µM OH-tamoxifen (T), or the combination (PT) for 72 hours assayed for viability. Vehicle and combination treated cells were further evaluated by western blot for PARP cleavage. For (**A**), (**B**), and (**D**) the average from three independent experiments is presented, with the error bars indicating the standard error of the mean. An (*) indicates a significant difference compared to vehicle treatment (P-value < 0.05), while an (#) indicates an insignificant difference (P-value > 0.05).

Our previous studies have shown that HDAC inhibitors (e.g. valproic acid, vorinostat, and entinostat) enhance the anti-tumor activity of tamoxifen in ER-positive breast cancer cells, by both reducing cell proliferation and increasing cell death [6,7]. However, the mechanism by which this combination therapy induces apoptosis is not fully understood. Several studies have demonstrated that HDAC inhibition reduces Akt activity. However, the inhibition of AKT activation alone is not sufficient to induce significant cell death. As the Akt pathway is a strong promoter of cell survival in breast cancer cells, we sought to evaluate the effect of HDAC inhibition on Akt in the presence of ER modulators. For pharmacological inhibition, we employed the hydroxamic acid-type HDAC inhibitor PCI-24781. PCI-24781 was chosen for use in this study for several key reasons, including its potency against HDAC2, which we have shown to be a crucial target for the efficacy of this therapeutic combination, and its relatively wide therapeutic window in patients [25,26]. ER positive MCF7 cells treated with tamoxifen and PCI-24781 exhibited a dose dependent increase in cell death with an increasing concentration of PCI-24781 (Figure 1A). As previously shown, treatment with either agent elicited a G1 cell cycle accumulation, primarily reducing the population of cells in S phase, and up regulation of the CDK inhibitor p21 (Figure S1). However, combined treatment did not result in a greater G1 population or p21 expression. Reduced viability was due to the induction of apoptosis, as evidenced by the substantial increase in the percentage of TUNEL-positive MCF7 cells with 0.1 µM PCI-24781 and 10 µM OH-tamoxifen co-treatment compared to control or single agent treatment (Figure 1B). Furthermore, western blot evaluation revealed elevated levels of the apoptotic drivers Bax and Bim, increased release of cytochrome C in the cytoplasm and PARP cleavage in MCF7 cells treated with the combination compared to untreated and single agent treated cells (Figure 1C). Evaluation of several ER-positive breast cancer cell lines (e.g. ZR75-1, BT474, and MDA361 cells) demonstrated that synergistic induction of apoptosis with the drug combination is not limited to MCF7 cells (Figure 1D).

**Figure 2:**
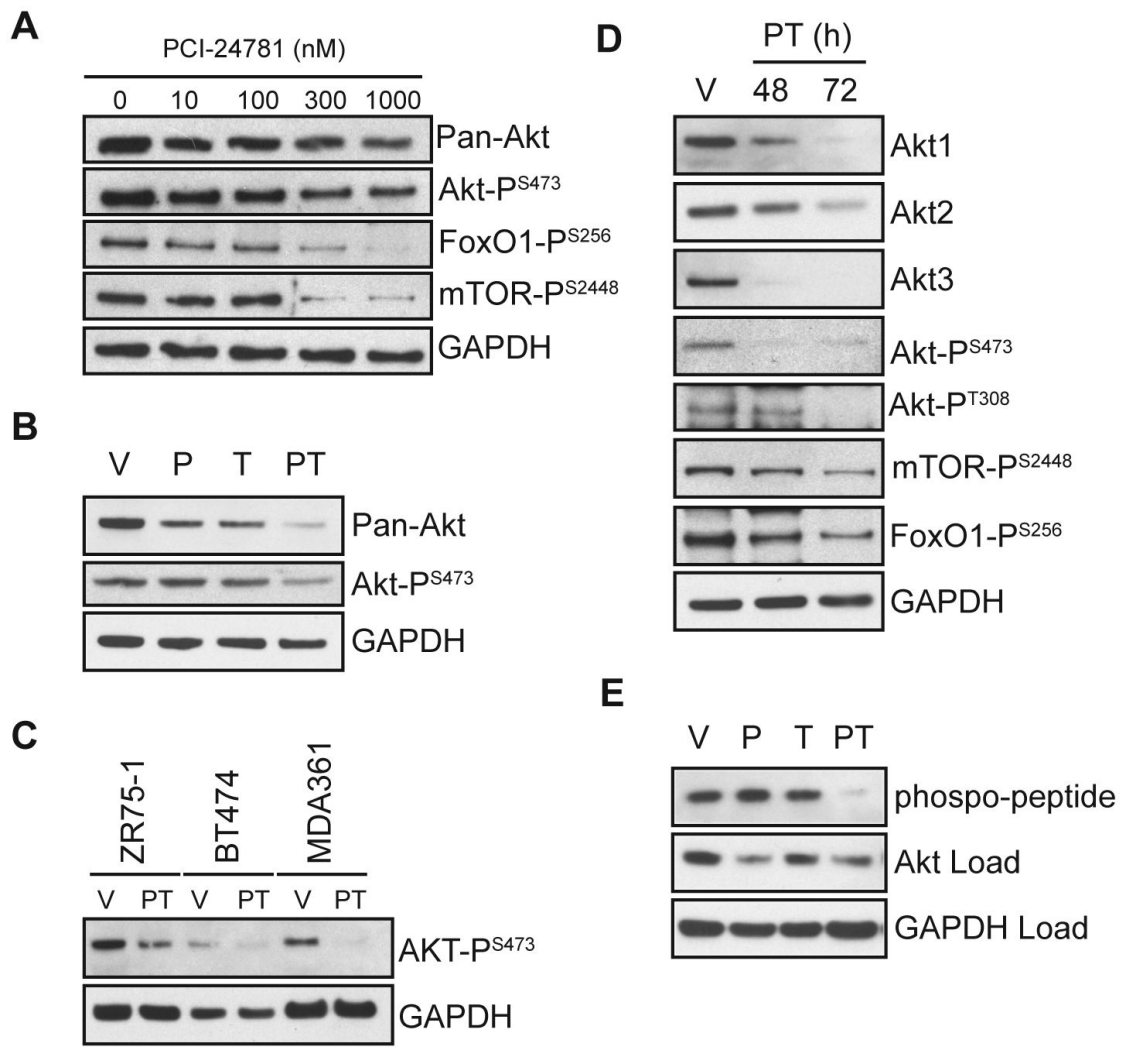
PCI-24781 and **tamoxifen concertedly down regulate Akt protein** and **activity in MCF7 cells.** (**A**) MCF7 cells were treated with increasing concentrations of PCI-24781 for 72 hours and western blotted. MCF7 cells were treated for 72 hours with vehicle (V), 0.1 µM PCI-24781 (P), 10 µM OH-tamoxifen (T), or the combination (PT) and cells extracts were evaluated by western blot (**B**) for Pan-Akt and active Akt (P^S473^) expression or (**E**) Akt activity *in vitro* by immunoprecipitating and then incubating phospho-Akt with a GSK-3 substrate and measuring levels of phosphorylated GSK-3. (**C**) The indicated cell lines were treated with vehicle (V) or 0.1 µM PCI-24781 and 10 µM OH-tamoxifen (PT) for 72 hours and western blotted for active Akt. (**D**) In cell extracts, levels of Akt isoforms, active Akt (P ^S473^and P^S308^) and down stream indicators of Akt activity following 48 and 72 hour treatment with 0.1 µM PCI-24781 and 10 µM OH-tamoxifen (PT) are compared to vehicle treated cells (V).

To evaluate the effect of PCI-24781 on Akt expression and activity, MCF7 cells were treated with increasing concentrations of PCI-24781 for 72 hours. Western blot analysis demonstrated reduced Akt expression and activity (P^S473^) and phosphorylation of down stream targets (FoxO1-P^S256^ and mTOR-P^S2448^) with increasing PCI-24781 concentrations (Figure 2A). To evaluate the combination of HDAC and ER inhibition on Akt, MCF7 cells were treated with vehicle, 0.1 µM PCI-24781, 10 µM OH-tamoxifen, or the combination for 72 hours and assayed by western blot (Figure 2B). Treatment with either PCI-24781 or OH-tamoxifen resulted in reduced Akt protein compared to vehicle treated cells. Akt protein was further reduced in cells treated with the combination (Figure 2B), affecting expression of all three Akt isoforms (e.g. Akt1, 2, and 3) (Figure 2D). However, a significant reduction in activated Akt (P^S473^) required both HDAC and ER inhibition (Figure 2B). This effect on activated Akt was conserved in several ER-positive breast cancer cell lines (Figure 2C). Over time, the reduction in phosphorylated Akt correlated with decreased phosphorylation of Akt target proteins (e.g. mTOR-P^S2448^ and FoxO1-P^S256^) (Figure 2D). To further determine whether a reduction in Akt protein and phosphorylation translated to reduced enzymatic activity, cell extracts were evaluated using an *in vitro* Akt kinase assay (Figure 2E). From cell extracts, phospho-Akt was immunoprecipitated and incubated with a GSK-3 substrate, and phosphorylated GSK-3 was measured by western blot. While Akt activity following treatment with either agent alone was comparable to vehicle treated control cells, treatment with the combination resulted in substantially less GSK-3 phosphorylation, consistent with the reduced Akt activation observed in cell extracts. Together these results demonstrate that HDAC inhibition and tamoxifen both reduce expression of Akt protein. However, only the concerted effort of both agents results in a substantive reduction in cellular Akt activity and increased cell death.

### HDACs regulate AKT mRNA levels

**Figure 3:**
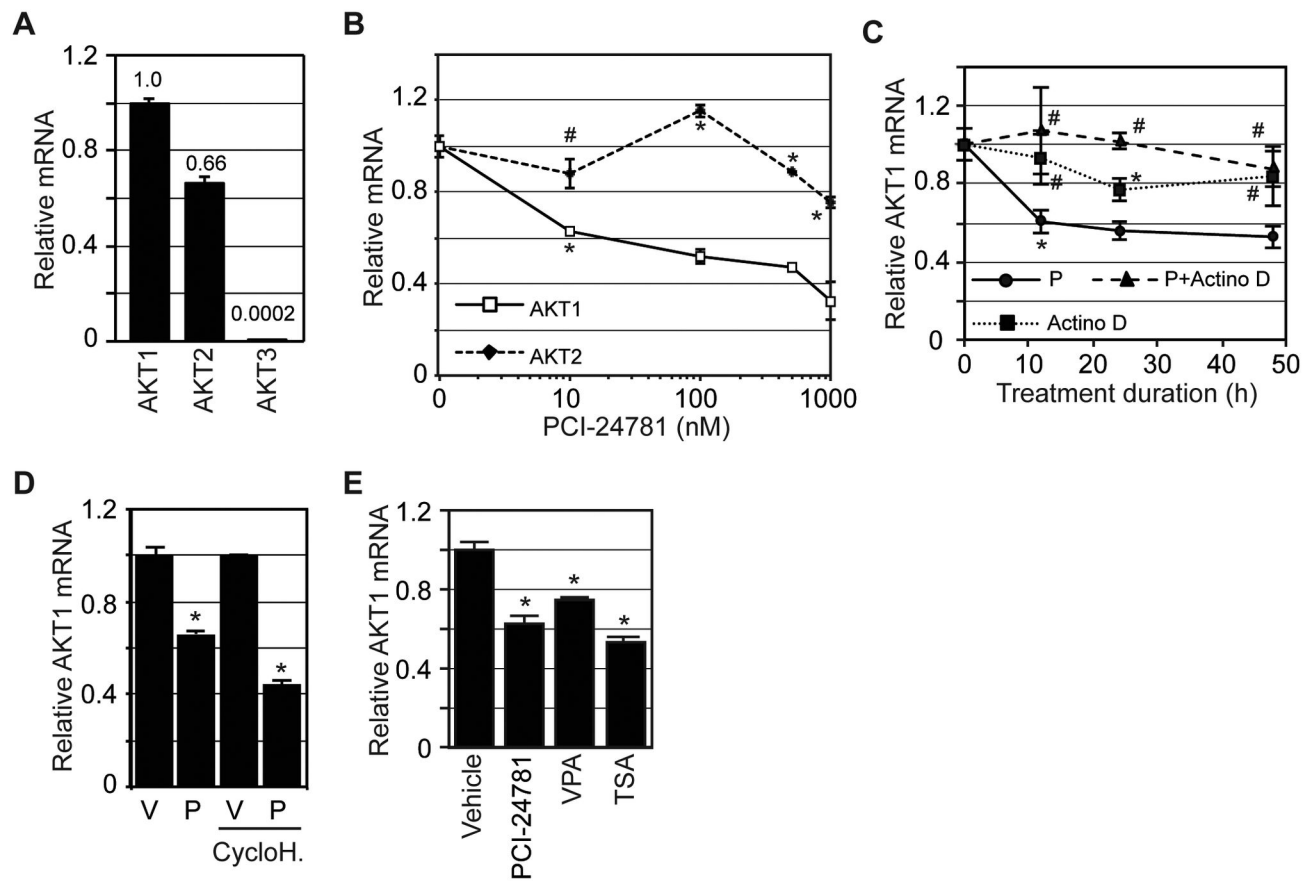
PCI-24781 regulates AKT mRNA levels. (**A**) Basal levels of AKT1, 2, and 3 mRNA in MCF7 cells are presented relative to AKT1 expression. (**B**) AKT1 and 2 mRNA expression was determined in MCF7 cells following treatment with increasing concentrations of PCI-24781 for 24 hours. (**C**) MCF7 cells were treated with 0.1 µM PCI-24781 (P), 5 µg/mL actinomycin D (Actino D), or the combination (P+Actino D) and evaluated for AKT1 mRNA expression after 0, 12, 24, and 48 hours treatment. (**D**) MCF7 cells were pretreated with vehicle or 10 µg/mL cycloheximide for 1 hour. Vehicle and cycloheximide pretreated cells were then each divided and treated with vehicle (V) or 0.1 µM PCI-24781 (P) with or without 10 µg/mL cycloheximide (CycloH) for 24 hours and evaluated for AKT1 mRNA expression. (**E**) MCF7 cells were treated with vehicle, 0.1 µM PCI-24781, 3 mM valproic acid (VPA), or 30 nM trichostatin A (TSA) for 24 hours and evaluated for AKT1 mRNA expression. All treatments were conducted in triplicate and expressed as the average with the error bars indicating the standard error of the mean. An (*) indicates a significant difference (P-value < 0.05) and a (#) an insignificant difference (P-value > 0.05) compared to vehicle or zero time treatment.

HDACs are essential components of co-regulatory complexes and key mediators of gene transcription. In addition, HDACs (e.g. HDAC6) can influence protein stability through relationships with chaperones such as HSP90 [27]. Previously, HSP90 inhibition was shown to result in reduced Akt protein stability [28]. Thus, the reduced Akt protein observed with PCI-24781 treatment may result from reduced transcription, protein stability, or a combination of both. Three Akt isoforms are expressed in breast cancer cells (e.g. Akt1, Akt2, and Akt3). Recently, relative abundance, rather than the intrinsic enzyme kinetics, was shown to determine how each isoform contributes to cellular Akt activity [29]. Evaluation of AKT isoform mRNA expression in MCF7 cells showed that AKT1 and 2 account for 60% and 40% of total cellular AKT mRNA respectively, while less than 0.1% is attributable to AKT3 (Figure 3A). As AKT1 and 2 are the most abundant isoforms, we sought to determine their relative mRNA expression following HDAC inhibition. MCF7 cells were treated with increasing concentrations of PCI-24781 for 24 hours and AKT1 and 2 mRNA levels were measured (Figure 3B). Addition of PCI-24781 reduced AKT1 mRNA expression by 40% with 10 nM and by 65% with 1 µM treatment. AKT2 mRNA expression remained relatively unchanged at clinically achievable doses (~0.1-0.2 µM), with a modest reduction (~20%) with 1 µM PCI-24781. Treatment over time showed that the effects plateaued by 12 hours (Figure 3C). To determine whether HDAC inhibition reduces AKT1 mRNA by inhibiting transcription or inducing degradation, MCF7 cells were treated with the transcriptional inhibitor actinomycin D and with or without PCI-24781 and evaluated over time (Figure 3C). The rate of AKT1 mRNA reduction was substantially less in the presence of both, actinomycin D and PCI-24781, than with either PCI-24781 or actinomycin D alone. This data suggests that PCI-24781 promotes AKT1 mRNA decay, as its reduction is greater than transcriptional inhibition. To assess whether this induced decay is direct or indirect resulting from the altered expression of another protein, MCF7 cells were first pretreated with the translation inhibitor cycloheximide for one hour, followed by the addition of vehicle or 0.1 µM PCI-24781 for 24 hours (Figure 3D). Treatment with cycloheximide did not rescue PCI-24781-induced loss of AKT1 mRNA, suggesting protein translation is not required for AKT1 down regulation. To ensure that the effect of HDAC inhibition on AKT1 mRNA expression is not specific to PCI-24781 treatment, MCF7 cells were treated with an alternative HDAC inhibitor of the same (trichostatin A, TSA) and of a different class (valproic acid, VPA). AKT1 mRNA expression was evaluated after a 24 hour exposure to 3 mM VPA or 30 nM TSA (Figure 3E). Both HDAC inhibitors significantly down regulated AKT1 mRNA, with TSA treatment eliciting a drop comparable to PCI-24781 treatment. Taken together, these results demonstrate that the HDAC inhibitor-mediated reduction of MCF7 Akt levels is in part due to decreased mRNA stability.

### PCI-24781 regulates AKT1 expression via an ER dependent mechanism

**Figure 4:**
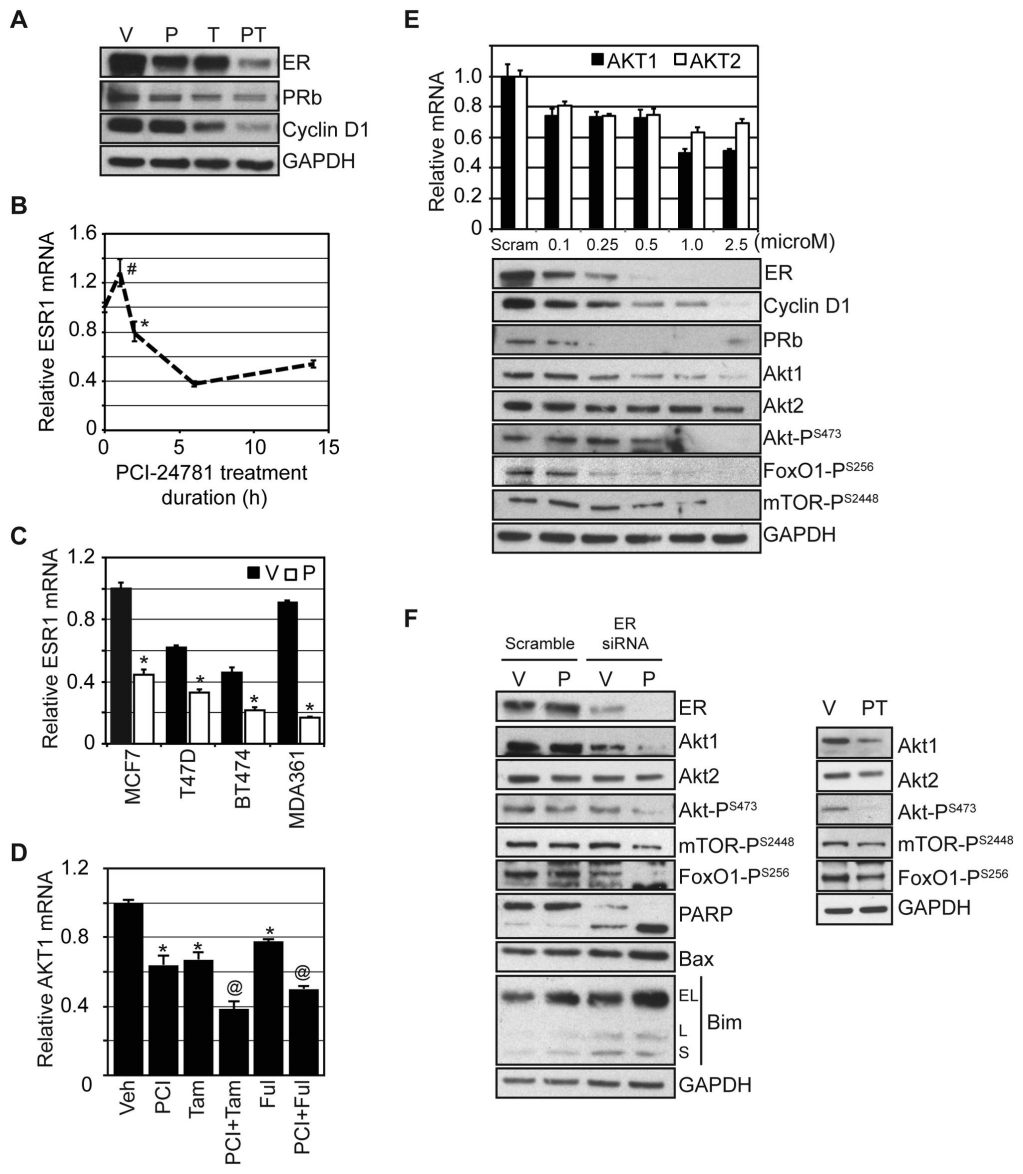
HDACs regulate AKT mRNA expression by an estrogen receptor-(α) dependent mechanism. (**A**) MCF7 cells were treated with vehicle (V), 0.1 µM PCI-24781 (P), 10 µM OH-tamoxifen (T), or the combination (PT) for 72 hours and western blotted. (**B**) MCF7 cells were treated with 0.1 µM PCI-24781 and ESR1 mRNA levels were measured at the indicated times and presented relative to untreated ESR1 levels. (**C**) The indicated cell lines were treated with vehicle (V) or 0.1 µM PCI-24781 (P) for 6 hours and ESR1 mRNA levels were measured and presented relative to vehicle treated MCF7 cells. (**D**) MCF7 cells were treated with vehicle (Veh), 0.1 µM PCI-24781 (PCI), 10 µM OH-tamoxifen (Tam), 0.1 µM PCI-24781 and 10 µM OH-tamoxifen (PCI+Tam), 0.1 µM fulvestrant (Ful), or 0.1 µM PCI-24781 and 0.1 µM fulvestrant (PCI-Ful) for 24 hours and AKT1 mRNA levels were measured and presented relative to vehicle treated MCF7 cells. (**E**) MCF7 cells were transfected with scramble or increasing concentrations ESR1 directed siRNA for 72 hours and assayed for AKT1 and AKT2 mRNA and western blotted for ER and Akt pathway components. AKT1 and AKT2 expression are normalized to individual scramble treatments and not to each other. For both AKT1 and 2, all ESR1 siRNA concentrations resulted in significant reductions (P-value < 0.05) compared to scramble transfection. (**F**) MCF7 cells were transfected with scramble or 1 µM ESR1 directed siRNA for 24 hours, divided and then treated with vehicle (V) or 0.1 µM PCI-24781 (P) for 72 hours and evaluated by western blot. For all mRNA measurements, experiments were conducted in triplicate and results expressed as the average with the error bars indicating the standard error of the mean. An (*) indicates a significant difference (P-value < 0.05) and a (#) an insignificant difference (P-value > 0.05) compared to vehicle or zero time treatment. A (@) indicates a significant (P-value < 0.05) difference compared to PCI-24781 treatment.

Previous work from our laboratory and others has shown that HDAC inhibitors regulate both the transcription of ER as well as its post-translational stability [6,30–32]. To confirm these findings with PCI-24781, we evaluated ER and ER response gene expression (e.g. PRb and Cyclin D1) in MCF7 cells following exposure to 0.1 µM PCI-24781, 10 µM OH-tamoxifen, or the combination (Figure 4A). Treatment of cells with OH-tamoxifen or PCI-24781 alone elicited a modest reduction of ER, PRb, and Cyclin D1 expression compared to vehicle treated cells, whereas the combination resulted in a substantial reduction of ER, PRb, and Cyclin D1 levels. ER mRNA (ESR1) decreased rapidly following 2 hours exposure to PCI-24781 and plateaued after six hours, reduced more than 60% (Figure 4B). A similar decrease in ESR1 expression following six hour treatment with PCI-24781 was observed in various ER-positive breast cancer cell lines, regardless of HER2 or PR status (Figure 4C).

Treatment with tamoxifen alone resulted in down regulation of Akt protein in MCF7 cells, but only in the presence of an HDAC inhibitor was Akt activity significantly reduced (Figure 2A). To determine whether the effects of HDAC inhibition on AKT expression and activity are mediated through ER signaling, MCF7 cells were treated for 24 hours with the ER modulator tamoxifen or the ER down regulator fulvestrant with and without PCI-24781 (Figure 4D). Both fulvestrant and tamoxifen down regulated AKT1 mRNA, which was further reduced when combined with PCI-24781. Treatment with either an anti-estrogen or siRNA-mediated depletion of ER had only a negligible effect on AKT2 expression (Figure S2). We therefore postulated that the synergistic interaction of an HDAC inhibitor and tamoxifen is mediated through AKT, which requires the inhibition of ER signaling. A role for ER in regulating AKT1 expression was supported by siRNA-mediated depletion of ER. Transfecting MCF7 cells with increasing concentrations of ESR1 directed siRNA resulted in a dose dependent decrease in expression of the ER response genes, PRb and Cyclin D1 (Figure 4E). Mimicking the effects on ER response genes, the siRNA depletion of ESR1 resulted in a dose dependent reduction of Akt levels and activity (Figure 4E). This data suggests that the HDAC inhibitor mediated effects on the ER are necessary to sufficiently reduce Akt activation and induce apoptosis. To test this, ESR1 was depleted in MCF7 cells and treated with either vehicle or 0.1 µM PCI-24781 for 72 hours (Figure 4F). Western blot analysis demonstrated that in cells depleted of ESR1 and treated with PCI-24781, Akt protein, activity, and signaling are down regulated, while pro-apoptotic Bim and Bax are up regulated and PARP cleavage is significantly increased. Together these results demonstrate that decreased AKT expression and activity are mediated through the ER. As PCI-24781 regulates ESR1 expression, it thus indirectly controls AKT1 expression through the ER. When combined with HDAC inhibition, depletion of the ER is sufficient to induce apoptotic cell death.

### AKT depletion is sufficient to enhance tamoxifen cytotoxicity

**Figure 5:**
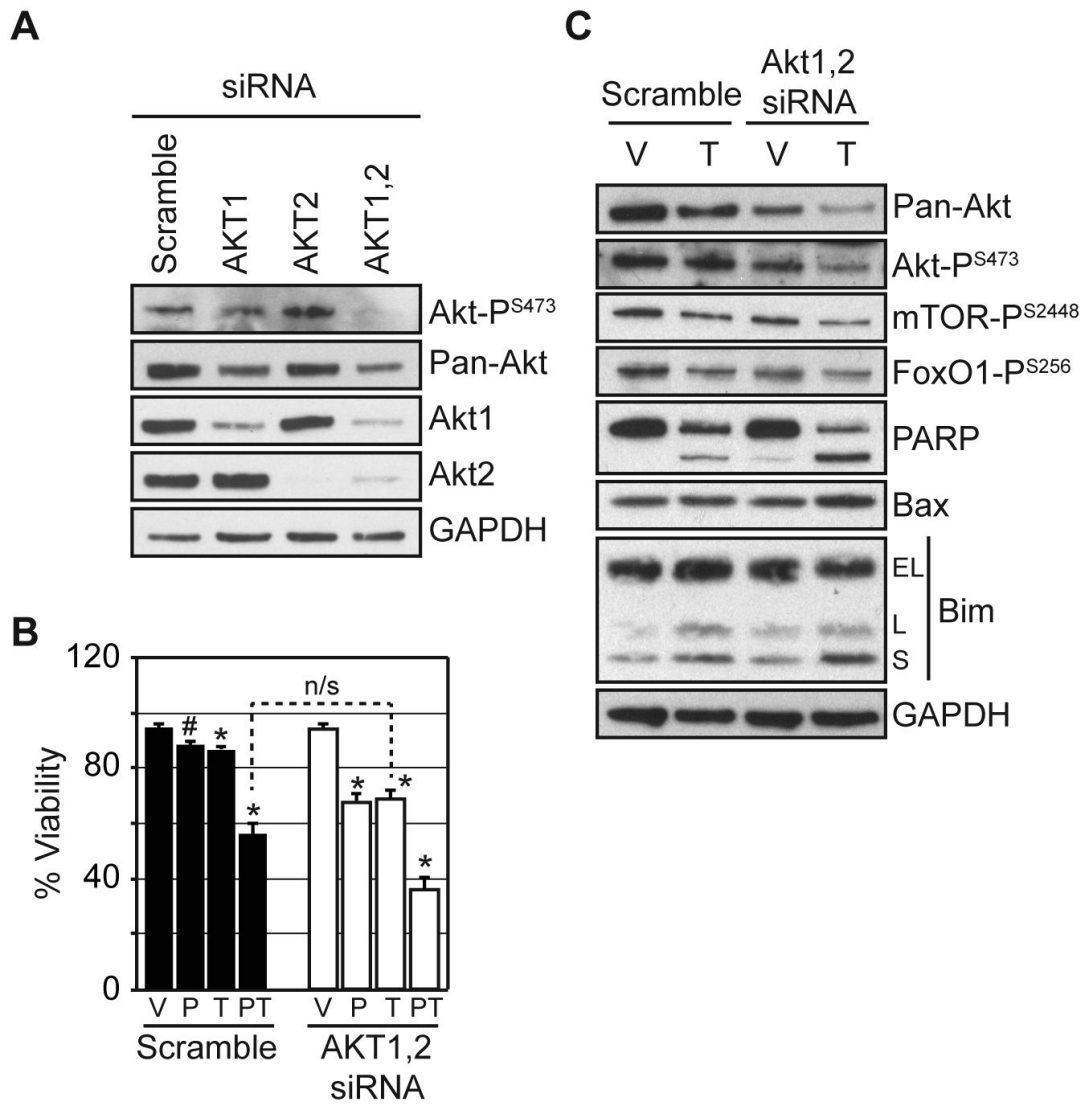
AKT depletion enhances tamoxifen cytotoxicity comparable to HDAC inhibition. MCF7 cells were transfected with 1 µM scramble, AKT1, AKT2, or AKT1 and 2 directed siRNA for (**A**) 48 hours and western blotted or (**B**) 24 hours followed by treatment with vehicle (V), 0.1 µM PCI-24781 (P), 10 µM OH-tamoxifen (T), or the combination (PT) for an additional 72 hours and evaluated for cell viability. (**C**) MCF7 cells were transfected with 1 µM AKT1 and 2 directed siRNA for 24 hours, then divided and treated with vehicle (V) or 10 µM OH-tamoxifen (T) for 72 hours and western blotted. Cell viability treatments were conducted in triplicate with results expressed as the average with the error bars indicating the standard error of the mean. An (*) indicates a significant difference (P-value < 0.05) and a (#) an insignificant difference (P-value > 0.05) compared to vehicle treatment. n/s indicates an insignificant difference between treatments (P-value > 0.05).

Akt is a major regulator of cell survival and proliferation. Our results demonstrate that Akt expression is regulated by HDACs. To further evaluate the connection between HDACs and the ER and their regulation of the Akt pathway, we sought to determine whether direct Akt depletion is sufficient to enhance tamoxifen cytotoxicity. AKT1 and 2 were depleted in MCF7 cells by introducing siRNAs to deplete both Akt1 and Akt2 individually as well as in combination using AKT directed siRNAs (Figure 5A). Akt1 depletion resulted in a greater reduction of total Akt compared to Akt2 depletion, as expected, due to the greater abundance of AKT1 in the cell (Figure 3A). Total Akt was further reduced with the combined AKT1 and 2 depletion. However, phosphorylated Akt (i.e. activated) was substantially reduced only when both Akt1 and 2 were depleted.

To evaluate the ability of AKT depletion to enhance tamoxifen cytotoxicity, MCF7 cells were transfected with either scramble or a combination of AKT1 and 2 directed siRNAs. After 24 hours, cells were treated with vehicle, 0.1 µM PCI-24781, 10 µM OH-tamoxifen, or the combination for an additional 72 hours and assayed for cell viability (Figure 5B). For cells transfected with scramble siRNA, either single agent treatment had a minimal effect on cell viability, while co-treatment reduced viability to 56%. Alone, depletion of AKT1 and 2 did not reduce viability compared to cells transfected with scramble siRNA and treated with vehicle. However, when AKT1 and 2 depletion was combined with either PCI-24781 or tamoxifen treatment, cell viability dropped to 67% and 69%, respectively, statistically comparable to scramble transfected cells treated with combined PCI-24781 and tamoxifen (P-value > 0.12 and 0.08, respectively). When AKT1 and 2 depletions were combined with PCI-24781 and tamoxifen co-treatment, cell viability was further reduced to 36%.

To determine whether Akt depletion in combination with tamoxifen elicited similar molecular changes to PCI-24781 and tamoxifen treatment, MCF7 cells were transfected with scramble or AKT1 and 2 directed siRNA overnight, divided, and treated with vehicle or 10 µM OH-tamoxifen for 72 hours. Following treatment, cell extracts were evaluated by western blot (Figure 5C). For cells receiving combined Akt depletion and OH-tamoxifen, Akt, activated Akt (e.g. P^S473^) and Akt signaling were down regulated. Furthermore, apoptosis was induced, as illustrated by increased cytotoxic Bim-s, Bax, and PARP cleavage.

Together these findings demonstrate that depleting AKT1 and 2 in MCF7 cells is sufficient to down regulate Akt activity, as seen with combined PCI-4781 and tamoxifen treatment. Furthermore, down regulation of AKT1 and 2 enhances the cytotoxicity of tamoxifen to an extent comparable to PCI-24781 treatment by inducing apoptosis through a similar mechanism. Thus, down regulation of AKT1 and 2 by combined PCI-24781 and tamoxifen treatment is likely a key component underpinning their cytotoxic efficacy.

## Discussion

A new therapeutic approach, combining HDAC inhibition with an anti-estrogen, for the treatment of breast cancer has been supported by promising preclinical and clinical studies. We have shown that addition of an HDAC inhibitor enhances the anti-tumor activity of tamoxifen by forcing tumor cells into apoptosis [6,7]. In a phase II trial, we evaluated the combination of the HDAC inhibitor vorinostat and tamoxifen for the treatment of women with heavily pretreated advanced breast cancer [9]. In a patient setting previously shown to no longer benefit from aromatase inhibitors or tamoxifen treatment alone [10], the addition of vorinostat resulted in durable responses and stable disease. In an effort to enrich for patients that would benefit from this novel therapy, we have sought to better characterize the underlying mechanism of this drug combination.

### HDACs and the ER regulate AKT mRNA

In this study, we demonstrate that HDAC inhibition in combination with anti-estrogens down regulates Akt mRNA, protein, and activity in breast cancer cells. Although previous works have shown that HDACs modulate the activation state of Akt post-translationally and its stability through HDAC6 and HSP90 [21,28], we show that HDACs further regulate AKT mRNA. HDAC inhibition primarily down regulates the most abundant isoform, AKT1. The transcriptional regulation of AKT by HDAC inhibitors is not influenced by the expression of ER, PR, or HER2. In MCF7 cells, HDAC inhibitor-mediated down regulation of AKT1 requires transcription, but not translation, suggesting that the effect of HDAC inhibition is direct. Additionally, HDAC inhibition results in greater AKT1 reduction than transcriptional inhibition alone, indicating reduced AKT mRNA is the result of promoting turnover. One mechanism consistent with these findings is microRNA-mediated degradation. An increase in microRNA expression would require transcription, but not translation, and promote mRNA degradation. In support of this possibility, HDACs have been demonstrated to regulate both the expression and maturation of microRNAs [33,34] and several microRNAs have been shown to control AKT1 and 2 mRNA levels [35–37]. However, depletion of the mircoRNA maturation protein DROSHA failed to rescue HDAC inhibitor-mediated down regulation of AKT mRNA (Figure S3). Furthermore, AKT1 mRNA exhibited unexpected biphasic decay overtime with PCI-34781 treatment. Whether this change in decay is intrinsic to the transcript, representing subpopulations that are differentially turned over, is the result of a cellular compensatory feed back loop, or the loss of drug potency is unknown and demands further study. Thus, it remains to be determined how HDACs regulate AKT mRNA and which HDACs are involved.

Several studies have argued that HDAC inhibition does not affect AKT expression, exerting its impact on the Akt pathway solely by regulating Akt phosphorylation and activation through the phosphatase PP1 [21,23]. However, these studies did not directly evaluate the effect of HDAC inhibition on the expression of AKT mRNA. To rule out a potential activity specific to PCI-24781, we demonstrated that AKT1 expression was reduced using both valproic acid and trichostatin A, the latter being used in the aforementioned studies. The observed inconsistency may also be the result of cell line differences, as one study utilized glioblastoma cells [21].

In addition to HDAC inhibition, inhibiting the ER, either with anti-estrogens such as tamoxifen and fulvestrant or by siRNA depletion, also down regulates AKT mRNA. As such, breast cancer cells that lack ER (e.g. MDA231 and SKBR3 cells) fail to exhibit AKT down regulation with anti-estrogen treatment (data not shown). When ER and HDAC inhibition are combined, an additive decrease in AKT1 expression is observed. As seen with PCI-24781, the influence of ER inhibition on AKT2 mRNA is limited, with a significant effect only seen in BT474 cells. It has been established in previous studies that HDACs regulate the expression and activity of the ER [38–40]. Not surprisingly, PCI-24781 down regulates ER mRNA, protein, and products of ER transactivation. As such, HDACs indirectly control AKT expression through an ER dependent mechanism.

### Concerted HDAC and ER inhibition is required for attenuated Akt activity and cytotoxicity

Individually, HDAC and ER inhibition down regulate AKT mRNA and together the effect is additive. The effect on Akt protein is comparable to its mRNA, decreasing with either agent alone and further reduced when combined. However, to achieve a significant reduction in Akt activity, treatment with both agents is required. The degree to which AKT mRNA reduction contributes to loss of Akt protein and activity is complicated by known roles for HDACs, where HDACs 1, 2, 3, and 6 are linked to the phosphorylation and activation of Akt and HDAC6 is further linked to Akt stability through its interaction with HSP90 [21–23,28]. The loss of Akt protein with combined HDAC and ER inhibition is likely the effect of reduced mRNA and protein stability. In MCF7 cells, neither PCI-24781 nor anti-estrogens significantly down regulate AKT2 or AKT3 mRNA. However, combined treatment with PCI-24781 and tamoxifen down regulates protein of all three Akt isoforms. Thus, reduced Akt2 and 3 proteins are likely the result of compromised HSP90 protein chaperone activity via HDAC6 inhibition. Consistent with this possibility, 0.1 µM PCI-24781 is sufficient to inhibit HDAC6 [41]. Furthermore, Basso et al. demonstrated in MCF7 cells that all three Akt isoforms exhibit increased ubiquitination and reduced expression when treated with the HSP90 inhibitor 17-AAG [28].

Several lines of evidence from the current study suggest that reduced AKT mRNA is sufficient to achieve reduced Akt activity and enhance tamoxifen cytotoxicity. A significant reduction in activated Akt is achieved when HDAC inhibition is combined with tamoxifen, the later of which is known only to affect AKT mRNA and not Akt protein stability or activation. Additionally, siRNA depletion of AKT1 and 2 resulted in a marked reduction of phosphorylated Akt. Depleting AKT1 and 2 enhanced the cytotoxicity of tamoxifen to levels comparable to those achieved with PCI-24781 and tamoxifen, down regulated Akt signaling, and promoted apoptosis by influencing expression of the same pro-apoptotic components (e.g. Bax and Bim). Thus, the depletion of AKT mRNA, resulting from combined HDAC and ER inhibition, may play a significant role in driving apoptotic cell death. Interestingly, depletion of AKT1 and 2 further increased cytotoxicity when combined with PCI-24781 or tamoxifen treatment, arguing that the addition of an Akt inhibitor to this combination may be a more effective strategy. This idea is supported by studies in other cancer types, including leukemia, head and neck squamous cell carcinoma, and non-small cell lung cancer, where cytotoxicity is enhanced, in some cases synergistically, when HDAC and Akt inhibition are combined [42–44].

## Conclusion

In the clinic, the combination of HDAC inhibition and anti-estrogen therapy for the treatment of breast cancer has yielded promising preliminary results. The ability to select patients with a greater likelihood to respond would represent a substantial improvement to this novel therapeutic approach. This study suggests that Akt is a key target, underpinning the effectiveness of this drug combination. Significantly, the down regulation of AKT mRNA or activity may represent a biomarker for predicting tumor response. Furthermore, these findings may be relevant to recent clinical studies showing promising benefits from the inhibition of the mTOR pathway in tumors of breast cancer patients treated with hormonal therapy [5,45].

## Supporting Information

Figure S1 PCI-24781 and tamoxifen delay cell cycle progression. (**A**) MCF7 cells were treated with vehicle, 0.1 µM PCI-24781 (PCI), 10 µM OH-tamoxifen (Tam), or the combination (PCI+Tam) for 24, 48, and 72 hours, stained with propidium iodide, and evaluated for cell cycle distribution using flow cytometry. (**B**) As treated in (**A**), MCF7 cells were harvested after 72 hours and western blotted for p21.
(TIFF)

Figure S2 Estrogen receptor inhibition does not significantly alter AKT2 expression in MCF7 cells. MCF7 cells were either treated with vehicle (C) or 100 nM fulvestrant (F) for 24 hours or transfected with scramble or ESR1 directed siRNA for 24 hours and assayed for AKT2 expression. Each treatment was conducted in triplicate and presented as the average. Error bars indicate the standard error of the mean. The fulvestrant treatment is normalized to the vehicle treatment, while the ESR1 siRNA treatment is normalized to scramble treatment. n/s indicates treatments are not significantly different (P > 0.5).
(TIFF)

Figure S3 DROSHA depletion does not rescue PCI-24781 mediated down regulation of AKT1. MCF7 cells were transfected with scramble or DROSHA directed siRNA for 48 hours, split and treated with vehicle (Veh) or 100 nM PCI-24781 (PCI) for 24 hours and assayed for AKT1 and DROSHA mRNA expression. Each treatment was conducted in triplicate and presented as the average. Error bars indicate the standard error of the mean. Both AKT1 and DROSHA expression was normalized to their respective vehicle treated scramble cells. (*) indicates a P-value < 0.5 compared to the vehicle treated scramble condition. (@) indicates a P-value < 0.5 compared to vehicle treated scramble condition. (#) indicates a P-value > 0.5 compared to vehicle treated scramble condition. ($) indicates a P-value < 0.5 compared to vehicle treated DROSHA siRNA condition.
(TIFF)
