# Supplementary figures and images for: Efficacy of Histone Deacetylase and Estrogen Receptor Inhibition in Breast Cancer Cells Due to Concerted down Regulation of Akt
Source: PLoS One. 2013 Jul 9;8(7):e68973. doi: 10.1371/journal.pone.0068973 (PMC3711340; doi:10.1371/journal.pone.0068973)

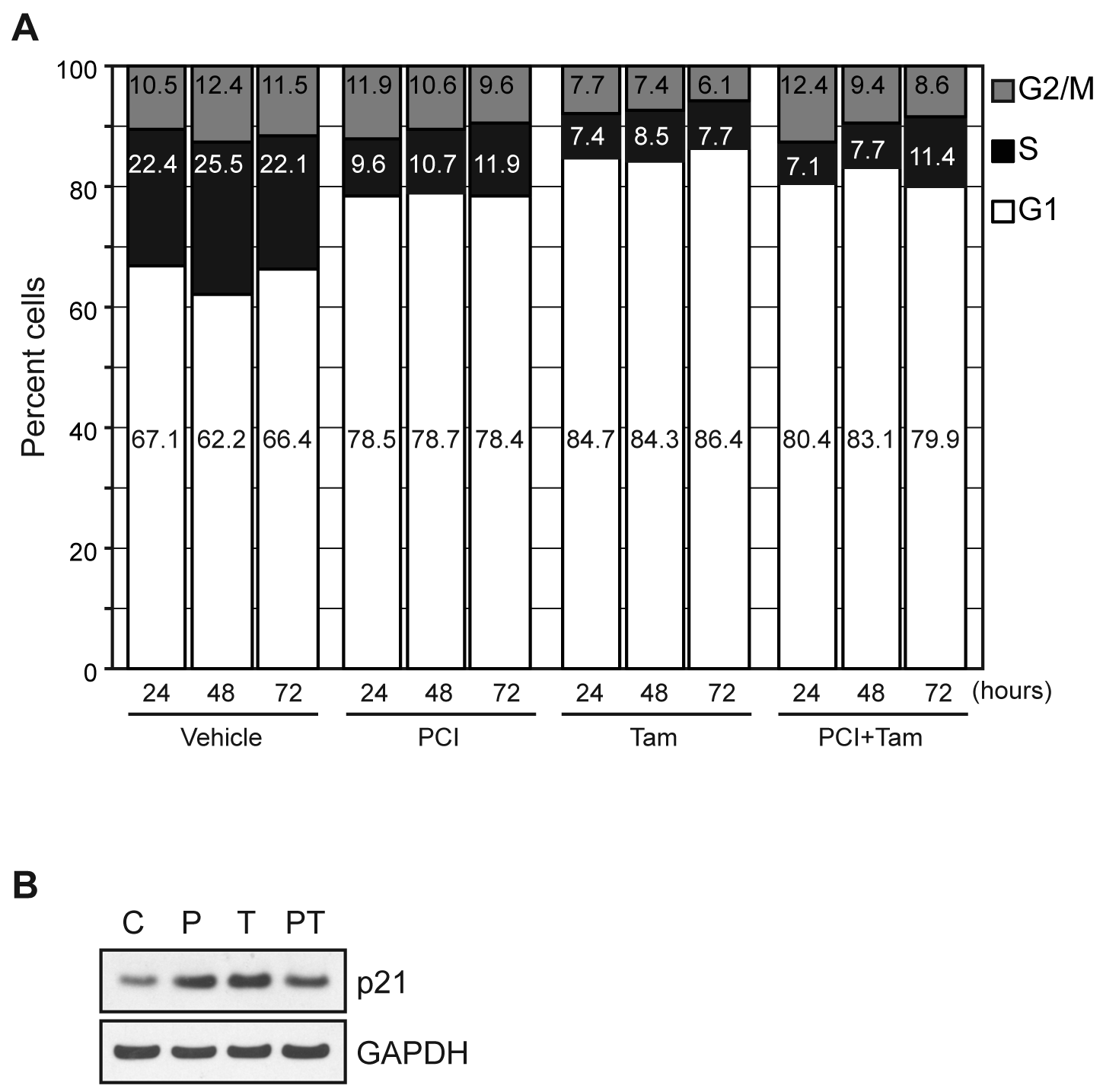

Supplement: Figure S1 — (A) MCF7 cells were treated with vehicle, 0.1 µM PCI-24781 (PCI), 10 µM OH-tamoxifen (Tam), or the combination (PCI+Tam) for 24, 48, and 72 hours, stained with propidium iodide, and evaluated for cell cycle distribution using flow cytometry. (B) As treated in (A), MCF7 cells were harvested after 72 hours and western blotted for p21. (TIFF) [file pone.0068973.s001.tiff]

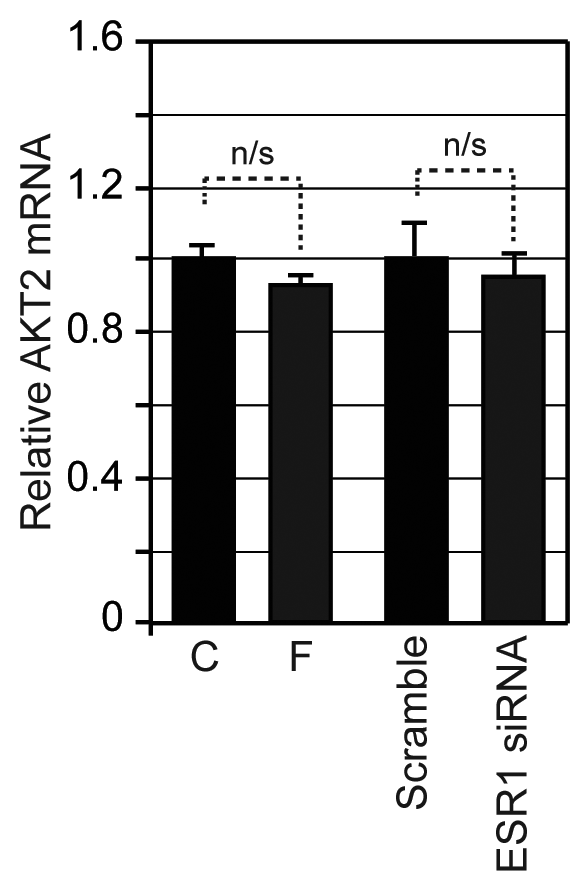

Supplement: Figure S2 — MCF7 cells were either treated with vehicle (C) or 100 nM fulvestrant (F) for 24 hours or transfected with scramble or ESR1 directed siRNA for 24 hours and assayed for AKT2 expression. Each treatment was conducted in triplicate and presented as the average. Error bars indicate the standard error of the mean. The fulvestrant treatment is normalized to the vehicle treatment, while the ESR1 siRNA treatment is normalized to scramble treatment. n/s indicates treatments are not significantly different (P > 0.5). (TIFF) [file pone.0068973.s002.tiff]

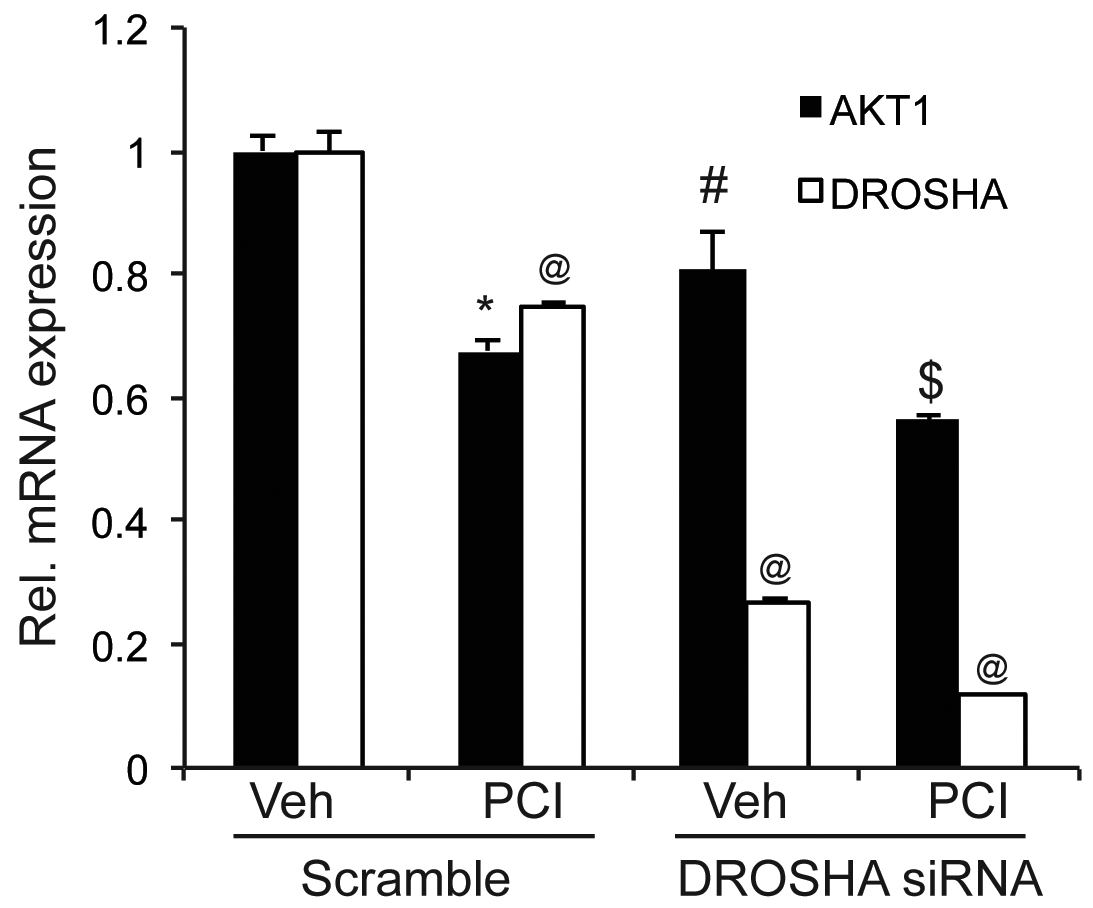

Supplement: Figure S3 — MCF7 cells were transfected with scramble or DROSHA directed siRNA for 48 hours, split and treated with vehicle (Veh) or 100 nM PCI-24781 (PCI) for 24 hours and assayed for AKT1 and DROSHA mRNA expression. Each treatment was conducted in triplicate and presented as the average. Error bars indicate the standard error of the mean. Both AKT1 and DROSHA expression was normalized to their respective vehicle treated scramble cells. (*) indicates a P-value < 0.5 compared to the vehicle treated scramble condition. (@) indicates a P-value < 0.5 compared to vehicle treated scramble condition. (#) indicates a P-value > 0.5 compared to vehicle treated scramble condition. ($) indicates a P-value < 0.5 compared to vehicle treated DROSHA siRNA condition. (TIFF) [file pone.0068973.s003.tiff]
